# Supplementary material for: HIVprotI: an integrated web based platform for prediction and design of HIV proteins inhibitors
Source: J Cheminform. 2018 Mar 9;10:12. doi: 10.1186/s13321-018-0266-y (PMC5845081; doi:10.1186/s13321-018-0266-y)
Supplement: Supplementary file 2 — Additional file 2. Source code of HIVProtI web server. [file 13321_2018_266_MOESM2_ESM.zip › HIVprotI_Source-code/hivprotI/weka-3-6-6/doc/weka/associations/AbstractAssociator.html]

AbstractAssociator


---


|  |  |  |
| --- | --- | --- |
| **Overview** \| **Package** \| **Class** \| **Tree** \| **Deprecated** \| **Index** \| **Help** \| **Weka's home** | |  |
| PREV CLASS   **NEXT CLASS** | **FRAMES**    **NO FRAMES**     **All Classes** |  |
| SUMMARY: NESTED \| FIELD \| CONSTR \| METHOD | DETAIL: FIELD \| CONSTR \| METHOD |  |


---


## weka.associations Class AbstractAssociator

```
java.lang.Object
  weka.associations.AbstractAssociator
```

**All Implemented Interfaces:**: java.io.Serializable, java.lang.Cloneable, Associator, CapabilitiesHandler, RevisionHandler

**Direct Known Subclasses:**: Apriori, FPGrowth, GeneralizedSequentialPatterns, PredictiveApriori, SingleAssociatorEnhancer, Tertius

---

``` public abstract class AbstractAssociator extends java.lang.Object implements java.lang.Cloneable, Associator, java.io.Serializable, CapabilitiesHandler, RevisionHandler ```

Abstract scheme for learning associations. All schemes for learning
associations extend this class

**Version:**
:   $Revision: 5503 $

**Author:**
:   Eibe Frank (eibe@cs.waikato.ac.nz)

**See Also:**: Serialized Form

---

**Constructor Summary**

`AbstractAssociator()`


| **Method Summary** | |
| --- | --- |
| `static Associator` | `forName(java.lang.String associatorName, java.lang.String[] options)`             Creates a new instance of a associator given it's class name and (optional) arguments to pass to it's setOptions method. |
| `Capabilities` | `getCapabilities()`             Returns the Capabilities of this associator. |
| `java.lang.String` | `getRevision()`             Returns the revision string. |
| `static Associator[]` | `makeCopies(Associator model, int num)`             Creates copies of the current associator. |
| `static Associator` | `makeCopy(Associator model)`             Creates a deep copy of the given associator using serialization. |

**Methods inherited from class java.lang.Object**

`equals, getClass, hashCode, notify, notifyAll, toString, wait, wait, wait`

**Methods inherited from interface weka.associations.Associator**

`buildAssociations`

**Constructor Detail**

### AbstractAssociator

```
public AbstractAssociator()
```


**Method Detail**

### forName

```
public static Associator forName(java.lang.String associatorName,
                                 java.lang.String[] options)
                          throws java.lang.Exception
```

:   Creates a new instance of a associator given it's class name and
    (optional) arguments to pass to it's setOptions method. If the
    associator implements OptionHandler and the options parameter is
    non-null, the associator will have it's options set.

    :   **Parameters:**: `associatorName` - the fully qualified class name of the associator: `options` - an array of options suitable for passing to setOptions. May be null. **Returns:**: the newly created associator, ready for use. **Throws:**: `java.lang.Exception` - if the associator name is invalid, or the options supplied are not acceptable to the associator

---


### makeCopy

```
public static Associator makeCopy(Associator model)
                           throws java.lang.Exception
```

:   Creates a deep copy of the given associator using serialization.

    :   **Parameters:**: `model` - the associator to copy **Returns:**: a deep copy of the associator **Throws:**: `java.lang.Exception` - if an error occurs

---


### makeCopies

```
public static Associator[] makeCopies(Associator model,
                                      int num)
                               throws java.lang.Exception
```

:   Creates copies of the current associator. Note that this method
    now uses Serialization to perform a deep copy, so the Associator
    object must be fully Serializable. Any currently built model will
    now be copied as well.

    :   **Parameters:**: `model` - an example associator to copy: `num` - the number of associators copies to create. **Returns:**: an array of associators. **Throws:**: `java.lang.Exception` - if an error occurs

---


### getCapabilities

```
public Capabilities getCapabilities()
```

:   Returns the Capabilities of this associator. Maximally permissive
    capabilities are allowed by default. Derived associators should
    override this method and first disable all capabilities and then
    enable just those capabilities that make sense for the scheme.

    :   **Specified by:**: `getCapabilities` in interface `Associator` **Specified by:**: `getCapabilities` in interface `CapabilitiesHandler`
    :   **Returns:**: the capabilities of this object **See Also:**: `Capabilities`

---


### getRevision

```
public java.lang.String getRevision()
```

:   Returns the revision string.

    :   **Specified by:**: `getRevision` in interface `RevisionHandler`
    :   **Returns:**: the revision


---


|  |  |  |
| --- | --- | --- |
| **Overview** \| **Package** \| **Class** \| **Tree** \| **Deprecated** \| **Index** \| **Help** \| **Weka's home** | |  |
| PREV CLASS   **NEXT CLASS** | **FRAMES**    **NO FRAMES**     **All Classes** |  |
| SUMMARY: NESTED \| FIELD \| CONSTR \| METHOD | DETAIL: FIELD \| CONSTR \| METHOD |  |


---
